# An epidemiological investigation on occurrence of enterohemorrhagic *Escherichia coli* in raw milk

**DOI:** 10.14202/vetworld.2018.1164-1170

**Published:** 2018-08-25

**Authors:** H. D. Vanitha, C. Sethulekshmi, C. Latha

**Affiliations:** Department of Veterinary Public Health, College of Veterinary and Animal Sciences, Mannuthy, Thrissur - 680 651, Kerala, India

**Keywords:** Enterohemorrhagic *Escherichia coli*, epidemiological investigation, epidemiological survey, multiplex polymerase chain reaction, raw milk

## Abstract

**Aim:**

The aim of the present investigation was to study the epidemiology of enterohemorrhagic *Escherichia coli* (EHEC) in raw milk and molecular characterization of isolates using multiplex polymerase chain reaction (PCR).

**Materials and Methods:**

A total of 125 raw milk samples were subjected to isolation, identification, and confirmation of virulence-associated genes by multiplex PCR (mPCR). The samples were collected from a milk cooperative society of Thrissur district, Kerala. For further epidemiological investigation, samples such as dung (126), hair coat of cow (60), udder swab (60), udder wash (60), milking utensil wash (36), Milker’s hand wash (36), water (36), soil (36), and feed (36) were collected from the households from which the raw milk tested positive for EHEC.

**Results:**

The occurrence of EHEC in individual raw milk samples was found to be 8.8%. The major source of contamination to raw milk was found to be dung (19.84%) followed by udder swab (16.67%), hair coat of cow (15%), Milker’s hand and milking utensils and water (11.11% each), and udder wash and soil (8.33% each). For identification of virulence genes, all the isolates were subjected to mPCR, of 75 isolates 73.33% of isolates harbored *stx* 2 gene while 53.33, 36, and 36% of isolates were encoded by *stx* 1, *eae* A, and *hly* A genes, respectively. On epidemiological survey, the multiple risk factors accountable for occurrence of EHEC in raw milk were found to be the quality of water used, improper and inadequate udder preparation, unhygienic hands of Milker’s, use of insufficiently cleaned milking utensils, and using common utensil for washings of udder and milking purposes.

**Conclusion:**

The result of the present study signifies that raw milk was contaminated with EHEC and possesses a high public health threat. As dairy cattle and its environment serve as a potential niche for EHEC, hygienic milking practices should be adopted to curb the occurrence of EHEC in raw milk.

## Introduction

Safe and nutritious food is the key to attain and promote good health. Besides this with a change in human food habits, the lifestyle foodborne diseases are showing an upward trend, resulting in high morbidity and mortality across the world. It has been reported that the diarrheal diseases contribute to more than half of global foodborne disease burden [1].

Among various foodborne pathogens, enterohemorrhagic *Escherichia coli* (EHEC) in particular EHEC O157:H7 is an important emerging pathogen responsible for gastrointestinal illnesses in children and elderly people. The very low infectious dose of EHEC ranging from 10 to 100 colony forming units makes them highly virulent and increases the risk of infection. In humans, EHEC cause infections ranging from mild diarrhea to life-threatening complications, namely hemorrhagic colitis and hemolytic uremic syndrome [2,3]. Milk is a highly nutritious and wholesome food, it forms an essential component of the human diet. India is the largest producer of milk and contributes to 18.5% of the world’s total production. In spite of greater achievements in terms of production, Indian dairy industry is lagging in exports due to the poor microbial quality of milk being produced. It is established that consumption of raw or contaminated milk serves as a potential mode of acquiring EHEC infection in humans. Cattle being reservoirs for EHEC carry these organisms in their intestine without any evident clinical illness. Furthermore, the pathogen can thrive well on various ecological niches on farm animals and their abiotic environment, namely on hair coat of animal, udder, cattle barn, water, feed, manure, and soil [4,5]. Hence, the risk from EHEC infection is a continuous challenge faced by the animal handlers. Moreover, the data regarding EHEC prevalence in dairy cattle rearing environment is largely unknown.

Various conventional culture techniques described for its isolation are time-consuming and are mainly based on the phenotypic characteristics. Polymerase chain reaction (PCR) based on genotypic characteristics are considered to be a rapid and promising technique for molecular confirmation of pathogens. Hence, multiplex PCR (mPCR) can be applied to food matrices as it aids in the rapid detection of multiple pathogens or multiple virulence factors of a single pathogen. *Escherichia coli* can be detected as well as differentiated into separate pathotype and serotypes using mPCR. Hence, application of mPCR is considered to be a rapid approach for early and easy detection of EHEC [6,7]. In EHEC infections, use of antibiotics is undesirable as it aggravates the release of verotoxins by the dying and dead bacterial cells [8]. Prevention of entry of pathogen to food forms more important criteria for reducing the risk of infection. Therefore, to ensure the safety of milk being produced and to curtail the occurrence of EHEC in raw milk, it is necessary to control the hazards throughout the production chain. This demands, data on the source of contamination and its mode of entry to milk.

The objective of the present research work was to study the epidemiology of EHEC in raw milk and retrospective trace backing of the source of contamination and route of entry into raw milk.

## Materials and Methods

### Ethical approval and Informed Consent

The present study did not involve any invasive procedure and hence no ethical approval is necessary. The samples were collected as per the standard collection procedure. However, animal owners who do not wish to participate were respected and not considered for the study. Confidentiality of the collected data was maintained. Verbal informed consent was obtained from each animal owner.

### Bacterial culture

The EHEC reference culture procured from the repository of Indian Veterinary Research Institute, Izzatnagar was used as a standard culture in the study.

### Collection of samples

The entire study was carried out in a milk cooperative society belonging to Thrissur district of Kerala. To select one milk cooperative society, pooled raw milk samples obtained from randomly selected milk cooperative societies belonging to Thrissur districts were screened for EHEC. From the selected milk cooperative society, positive milk samples were subjected to epidemiological study.

In the selected cooperative society of total 230 cattle, 125 cattle were in lactation, and hence 125 raw milk samples were collected from the society and were screened for the presence of EHEC. To determine the source of contamination and route of entry of EHEC into raw milk an epidemiological study was performed in each of the EHEC positive households. The following samples were collected at the time of milking from the animal such as dung (126), hair coat of cow (60), and udder swab (60). The samples were also collected considering the milking practices and environmental parameters existing in and around the household premises which included udder wash (60), milking utensil wash (36), Milker’s hand wash (36), water (36), soil (36), and feed (36) samples. The above-mentioned samples were collected from all the positive houses for 6 times at an interval of 2 weeks and examined for the presence of EHEC.

All the samples were collected during the study period from August 2016 to May 2017. The aseptically collected samples were brought to the laboratory under the refrigerated condition and processed for the analysis within 24 h.

### Epidemiological survey

An epidemiological survey was conducted through a well-designed questionnaire to know the various risk factors involved in milking practices and their influence on occurrence of EHEC in raw milk samples. At the time of milk sample collection, a total of 92 farmers were interviewed, and the data on hygienic milking practices adopted by the farmers such as hand, udder, and milking utensil hygiene practices that had an indirect bearing on the quality of milk were collected.

### Preparation of the collected samples

All the collected samples were processed in Quality Control Laboratory, Department of Veterinary Public Health, College of Veterinary and Animal Sciences, Thrissur. About 25 g of solid samples were transferred into 225 ml of trypticase soy broth (TSB) and homogenized for 180 s (Smasher, AES, France). The 25 ml liquid samples were pre-enriched in 225 ml of TSB while swab samples were pre-enriched in 45 ml of TSB.

### Isolation and cultural characteristics of EHEC

Samples were subjected to isolation and identification of EHEC as per Meng *et al*. [9] with certain modifications. 1 mL of pre-enriched sample was transferred to EC broth for selective enrichment at 37°C for 24 h. A loopful of the enriched sample was streaked onto eosin methylene blue (EMB) agar and incubated overnight at 37°C. For selective isolation of EHEC, three typical *E. coli* colonies with a metallic sheen on EMB agar were streaked onto Cefixime Tellurite-Sorbitol MacConkey agar (CT-SMAC) supplemented with novobiocin, cefixime, and potassium tellurite followed by incubation at 37°C for 18 h. To detect β-glucuronidase activity, the isolates were streaked onto 4-methylumbelliferyl β D-glucuronide (MUG EC) agar as described by Fujisawa *et al*. [10]. Non-fluorescent colonies observed under UV transilluminator were further subjected to primary and secondary biochemical tests.

### Molecular characterization of isolates using mPCR

**Figure-1:**
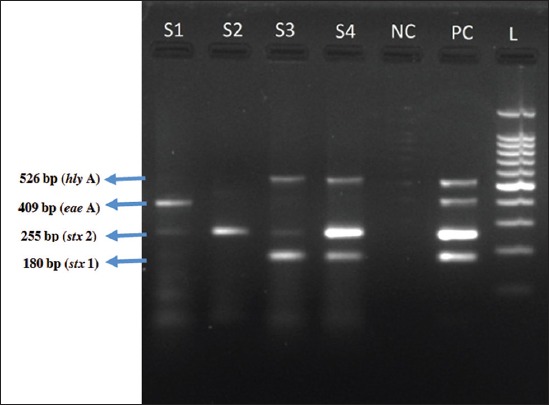
Multiplex polymerase chain reaction. S1-S4: Enterohemorrhagic *Escherichia coli* (EHEC) isolates from samples, NC: Negative control, PC: Positive control (EDL 933 strain of EHEC), L-100 bp ladder (180 bp – *stx* 1, 255 bp – *stx* 2, 409 bp- *eae* A, and 526 bp – *hly* A gene).

A mPCR was standardized against virulence genes of EHEC using EDL 933 strain of *E. coli* O157:H7. Virulence gene profile of the isolated EHEC strains was analyzed using mPCR with primers specific against *stx* 1, *stx* 2, *eae* A, and *hly* A genes, producing amplicons of size 180, 255, 409, and 526 bp, respectively (Figure-1). The primer for detection *eae* A gene was designed in the present study based on the sequence available at National Center for Biotechnology Information database operon (accession code: CP017446.1). The primers used and their corresponding product size are enlisted in Table-1 [11,12].

**Table-1:** Primer sequences and their respective product sizes.

Genes	Primer	Primer sequence	Size (bp)	References
*stx* 1	F	5’ATAAATCGCCATTCGTTGACTAC3’	180	[12]
	R	5’- AGAACGCCCACTGAGATCATC3’		
*stx* 2	F	5’-GGCACTGTCTGAAACTGCTCC3’	255	[12]
	R	5’-TCGCCAGTTATCTGACATTCTG3’		
*hly* A	F	5’-AGCCGGAACAGTTCTCTCAG3’	526	[11]
	R	5’-CCAGCATAACAGCCGATGT3’		
*eae* A	F	5’-ACGGTCTGGATCGTATCGTC3’	409	Designed in the present study
	R	5’-GCATCCGTTTTGGCACTATT3’		

The bacterial DNA was subjected to mPCR with a reaction volume of 30 µl consisting of 2.5 µl template, 3 µl 10× PCR buffer (200 mM), 2 µl MgCl_2_ (25 mM), 2.5 µl dNTP mix (2 mM each), 0.75 µl taq DNA polymerase (5 units/µl), 1 µl each forward and reverse primers of *stx* 1, *stx* 2, and *eae* gene, and 0.5 µl primers of *hly* A gene. The final volume was adjusted to 30 µl with nuclease-free water. The mPCR cycle consisted of initial denaturation at 95°C for 5 min followed by 35 cycles of denaturation at 95°C for 35 s, annealing at 60°C for 45 s, and elongation at 72°C for 2 min. The final extension was carried out for 10 min at 72°C. The primers used and their expected product size are enlisted in Table-1. The PCR products were separated on 1.5% agarose gel stained with ethidium bromide.

### Statistical analysis

The data obtained were statistically analyzed using the SPSS version 21.0. Since the sample size was more than 30, the occurrence of EHEC between various samples collected from dairy cattle rearing environment was compared using Z-test. The differences between frequencies or proportions in the occurrence of virulence genes in EHEC isolates were tested by Cochran’s Q-test. Fisher’s exact test and Z-test were used to study the influence of various risk factors involved in milking practices followed by farmers on occurrence of EHEC in raw milk.

## Results

### Occurrence of EHEC in raw milk samples

Out of 125 raw milk samples analyzed, 8.8% of the samples were contaminated with EHEC (Table-2). Of the 11 isolates obtained, *stx* 1, *stx* 2, *eae* A, and *hly* A genes were detected in 27.27, 90.91, 18.18, and 27.27% of isolates. Statistical analysis using Cochran’s Q test revealed a significant difference (p<0.05) in occurrence of *stx* 2 gene from that of all the other virulence genes among isolates from raw milk (Table-3).

**Table-2:** Overall, the occurrence of EHEC in raw milk and various samples from dairy cattle rearing environment.

Samples	Total samples analyzed	EHEC positive samples	

		Number	%
Raw milk	125	11	8.8
From dairy cow			
Dung	126	25	19.84^a^
Hair coat of cow	60	9	15.00^ab^
Udder swab	60	10	16.67^ab^
From milking practices			
Udder wash	60	5	8.33^b^
Milking utensil wash	36	4	11.11^ab^
Milker’s hand wash	36	4	11.11^ab^
From dairy environment			
Water	36	4	11.11^ab^
Soil	36	3	8.33^ab^
Feed	36	0	0
Total	611	75	12.27

Figures bearing at least one common superscript do not differ significantly (p<0.05). EHEC=Enterohemorrhagic *Escherichia coli*

**Table-3:** Occurrence of EHEC virulence genes (%) in different samples.

Samples	Total samples analyzed	Total isolate	Distribution of virulence genes (%)			

			*stx* 1	*stx* 2	*stx* A	*hly* A
IM	125	11	27.27^a^	90.91^b^	18.18^a^	27.27^a^
D	126	25	80^a^	68^ac^	40^bc^	36^b^
HC	60	9	44.44^a^	77.78^a^	44.44^a^	33.33^a^
US	60	10	40^ac^	80^bc^	30^ac^	0^a^
UW	60	5	80^a^	80^a^	40^a^	20^a^
MUW	36	4	25^a^	75^a^	0a	75^a^
MHW	36	4	50^a^	75^a^	50^a^	75^a^
W	36	4	50^a^	50^a^	50^a^	50^a^
S	36	3	0^a^	33.33^a^	66.67^a^	66.67^a^
F	36	0	0	0	0	0
Total	611	75	53.33	73.33	36	36

Figures bearing the same superscript within the row do not differ significantly (p<0.05). IM=Individual raw milk, D=Dung, US=Udder swab, UW=Udder wash, MUW=Milking utensil wash, MHW=Milker’s hand wash, W=Water, S=Soil, F=Feed, EHEC=Enterohemorrhagic *Escherichia coli*

### Occurrence of EHEC in different samples from dairy cattle rearing environment

Of all the samples analyzed, EHEC was isolated from 19.84, 16.67, and 15 % of dung, udder swab, and hair coat samples, respectively. The 11.11 % of milking utensil wash, Milker’s hand wash, and water samples were contaminated with EHEC. Whereas the occurrence of EHEC in udder wash and soil samples was found to be 8.33%. None of the feed samples examined were contaminated with EHEC (Table-2). Overall, the occurrence of EHEC in samples of dairy cattle rearing environment was found to be 12.27%. Dung was identified as the major source of contamination to raw milk followed by udder, hair coat, milking utensil, Milker’s hand, and water and soil samples.

The statistical analysis of data using Z test revealed that occurrence of EHEC in dung samples differed significantly (p<0.05) from that of udder wash samples. However, occurrence of EHEC in all the other samples examined from dairy cattle rearing environment did not differ significantly (p<0.05) (Table-2).

Cochran’s Q test revealed a significant difference (p<0.05) in the occurrence of *stx* 1 and *hly* A gene in isolates from dung samples while the isolates from udder swab differed significantly with respect to occurrence of *stx* 2 and *hly* A gene (Table-3).

On mPCR all the 75 isolates obtained by conventional culture technique, coded either of virulence genes (Figure-1). Among the isolates recovered, occurrence of *stx* 2 gene was more predominant (73.33%) against *stx* 1 gene (53.33%) while *hly* A and *eae* A genes were detected in 36% of isolates. The distribution of virulence genes among the positive isolates from different sources is as shown in Table-3.

### Epidemiological survey

Association of hygienic milking practices followed by farmers with the occurrence of EHEC was studied using a well-designed questionnaire, and the details are given in Table-4. All the farmers in the study milked their animals twice daily and were small-scale holders. It was noticed that the highest occurrence of EHEC was seen in the milk samples obtained from stall-fed cattle than the grazed cattle. Among the grazed cattle more number of EHEC were recovered from the milk samples of those animals that were grazed within premises against those that were let on to the common pasture for grazing.

**Table-4:** Association of various risk factors involved in milking management practices with the occurrence of EHEC in raw milk.

Management practices	Types	Frequency (%) N=92	The occurrence of EHEC in raw milk	

			Number	%
Feeding pattern	Grazing	17 (18.48)	2	13.33^a^
	Stall feeding	25 (27.17)	4	16.00^a^
	Both grazed and stallfed	50 (54.35)	4	8.00^a^
Grazing field	Grazed on premises	36 (39.13)	4	11.11^a^
	Grazed on common pasture	56 (60.87)	6	10.71^a^
Source of water	Open well	66 (71.74)	5	7.58^a^
	Pond	14 (15.22)	2	14.29^a^
	Bore well	12 (13.04)	3	25.00^a^
History of diarrhea	Yes	42 (45.65)	10	23.81
	No	50 (54.35)	0	0.00
Washing of udder before milking	Water	75 (81.52)	10	13.33
	Antiseptic solution	17 (18.48)	0	0.00
Washing of Milker’s hands before milking	Water	72 (78.26)	10	13.89
	Soap water	20 (27.74)	0	0.00
Type of milking utensils used	Widemouthed	34 (36.96)	4	11.76^a^
	Narrow mouthed	58 (63.04)	6	10.34^a^
Method of washing of milking utensils	Water	34 (36.96)	7	20.59^a^
	Hot water	29 (31.52)	2	6.90^ab^
	Using detergent	29 (31.52)	1	3.45^b^
Use of the same vessel for washing of udder and milking purpose	Yes	47 (51.09)	10	21.28
	No	45 (48.91)	0	0.00
Manure management	Composting	24 (26.09)	4	16.67^a^
	Agriculture use	68 (73.91)	6	8.82^a^
Distance between manure pit and milking area	<2 m	43 (46.74)	6	13.95^a^
	2–5 m	33 (35.87)	2	6.06^a^
	>5 m	16 (17.39)	2	12.5^a^

Figures bearing the same superscript between rows within each management practices do not differ significantly (p<0.05). EHEC=Enterohemorrhagic *Escherichia coli*

It was observed that isolation rate of EHEC in raw milk was highest among the households using plain water for washing of hand, udder, and milking utensils against those households who used an antiseptic solution such as potassium permanganate solution. The raw milk from the houses wherein the same milking utensil was used for washing of udder and milking purposes were highly contaminated with EHEC. Hence, the major risk factors governing the occurrence of EHEC in raw milk were identified as the health status of animal, contaminated udder, unhygienic milking utensil, and Milkers’ hands.

## Discussion

Milk is considered to be sterile when secreted from the udder of an apparently healthy animal. Thereafter, it is prone to contamination with pathogenic bacteria at various stages from production until consumption. Raw milk consumption is still practiced by some sector of population with an assumption that nutritional qualities and health benefits will be higher for raw milk than the boiled or processed milk which intensifies the risk of acquiring EHEC infection.

From this study, it is evident that raw milk serves as a potential source of EHEC as it was isolated from 8.8% of samples. Similarly, in the previous studies Neher *et al*. [13], Garbaj *et al*. [3], and Sethulekshmi and Latha [14] had isolated STEC from 13.7, 11, and 7.5% of milk samples from Guwahati, Libya, and Thrissur district of Kerala, respectively. Much lower occurrence of EHEC in raw milk samples was described by Solomakos *et al*. [15] and Ntuli *et al*. [16] who reported that 2.2 and 3% of samples were contaminated with EHEC. This may be due to differences in the prevalence of pathogen in the animals of different region and the milking practices adopted by the farmers.

Among the different samples collected from dairy cattle rearing environment, the highest occurrence of EHEC was seen among the dung samples indicating that the dairy cattle can serve as reservoir and void EHEC inconsistently. Similar findings were reported by Cobbold *et al*. [17] wherein 20% of dairy cattle were fecal carriers for EHEC. The pathogens can survive for a longer period in cattle manure [18]. Hence, regular and thorough cleaning of shed is essential to prevent contamination of cattle shed environment.

Other sources of contamination to raw milk were externally contaminated udder, hair coat of cow, milking utensil, Milker’s hand, and water and soil samples. Similarly, Abdissa *et al*. [19] and Anu [20] had isolated EHEC from 0.54 and 2.65% of hair coat samples while Fremaux *et al*. [21] and Msolo *et al*. [4] found that 30.86 and 55% cattle udder were contaminated with EHEC. In the present investigation, the occurrence of EHEC in the hair coat and udder swabs was higher as the cattle were shedding these organisms in feces and that might have contributed the pathogen onto the surface of udder and hair coat under poor housing condition. In all the houses studied, the floors of cattle barn were improper with crates and crevices where the manure can settle and that hindered the cleaning procedure. In a study, Nanu *et al*. [22] reported that udder and teat wash harbored EHEC. Hence, thorough and complete washing of udder followed by complete drying using a clean towel is an essential step to obtain milk free from contamination.

The occurrence of EHEC in milking utensil rinses and Milker’s hand washes proved that insufficiently cleaned milking utensils and Milker’s hands contribute various microorganisms into raw milk. In 2016, Msolo [23] had reported a higher prevalence of EHEC O157:H7 in milking machine swabs (50%) and hand swabs of Milker’s (33.33%). This could be attributed to improper washing of hands and poor hygiene of milking utensils.

Results of the present study also proved that the samples from dairy environment such as soil and water were contaminated with EHEC. While results were not in accordance with Wetzel and LeJeune [24] who recorded higher prevalence of EHEC in farm water samples (67%) against Ateba and Mbewe [25] and Halabi *et al*. [26] who reported a lower prevalence of 2.3 and 3.9%, respectively. Polifroni *et al*. [5] had isolated EHEC from 37% of soil samples from dairy farm environment against Parul *et al*. [27] who could not isolate EHEC from the soil samples. Hence, cross-contamination of water and soil with dung should be taken care to protect the raw milk from contamination. Potable water should be used for washing of udder, hands, and utensils before milking to ensure the safety of milk. None of the concentrate feed samples analyzed in this study harbored EHEC as the concentrate feed was stored away from the shed. Similar observations were recorded by Das *et al*. [28], while; Polifroni *et al*. [5] had isolated EHEC from 18 % of feed samples in Argentina.

Among the isolates recovered, the frequency of detection of *stx* 2 was higher followed by *stx* 1, *eae* A, and *hly* A gene. Detection of virulence genes confirmed the sensitivity of conventional culture technique. In 2006, Fremaux *et al*. [21] detected *stx* genes in 80 fecal and 38 environmental isolates from French dairy farm. The author reported a similar trend in the distribution of virulence genes as observed in this study where *stx* 2 (84%) was frequently detected followed by *stx* 1 and *eae* A genes (39 and 18%, respectively).

The highest occurrence of EHEC among those cattle that were grazed within premises can be attributed to the contaminated pasture where the manure was used for agriculture purpose within the premises. Similarly, Hancock *et al*. [29] have found that the prevalence of EHEC was more among those cattle that were grazed on manure applied pasture (85%) when compared that animal that was maintained on feedlot (82%). A high number of EHEC isolates were observed among the households who used only water for washing of udder and hands before milking than those who used antiseptic solution. This was in agreement with an India study and an Ethiopia study carried out by Neeta *et al*. [30] and by Tegegne and Tesfaye [31], respectively, who found that use of antiseptic agent for washing of hand and udder significantly affected the microbial quality of raw milk.

## Conclusion

The results of the present epidemiological investigation on occurrence of EHEC in raw milk samples collected from cooperative society belonging to Thrissur district of Kerala indicated that raw milk was contaminated at primary production level itself. The major risk factors governing the occurrence of EHEC in raw milk were identified as the health status of animal, contaminated water, soil, udder, unhygienic milking utensil, and Milkers’ hands. Presence of *E. coli* and EHEC in raw milk samples possess a great health risk to raw milk consuming population. Detection of both the primary virulence factors (Shiga toxin-encoding gene) and associated virulence factors (intimin and hemolysis) showed that dairy environment serves as a potential niche for EHEC and the dairy farmers were continuously exposed to this pathogen. Hence, this study demands a nationwide education of farmers regarding hygienic milking practices and its benefits in assuring the safety of raw milk to the public. Furthermore, awareness should be created among the dairy farmers on various zoonotic disease transmissions from dairy environment and the preventive measures.

## Authors’ Contributions

VHD collected the samples, carried out the laboratory work. SC and LC supervised the research work, reviewed the manuscript, and guided research work. VHD, SC, and LC contributed to the molecular work of the study and revised the manuscript. All authors read and approved the findings of the manuscript.
